# Estimating dispersal rates of Steller sea lion (*Eumetopias jubatus*) mother-pup pairs from a natal rookery using mark-resight data

**DOI:** 10.1371/journal.pone.0189061

**Published:** 2017-12-06

**Authors:** Carey E. Kuhn, Kathryn Chumbley, Lowell Fritz, Devin Johnson

**Affiliations:** Marine Mammal Laboratory, Alaska Fisheries Science Center, National Marine Fisheries Service, National Oceanic and Atmospheric Administration, Seattle, Washington, United States of America; Institute of Animal Science, CZECH REPUBLIC

## Abstract

To monitor population trends of Steller sea lions (*Eumetopias jubatus*) in Alaska, newborn pups are counted during aerial surveys. These surveys are scheduled to occur after the majority of pups are born, but before pups begin to spend significant time in the water. Some studies have reported dispersal of mother-pup pairs away from breeding beaches during the pupping season (July), which may influence survey results. Using a multistate mark-recapture model with state uncertainty, we estimated the amount of dispersal during the pupping season based on observations of permanently marked sea lions. Research was conducted at land-based observation sites on Marmot Island, Alaska, between 2000 and 2013. Both marked adult females with dependent pups and marked pups were observed at two rookery beaches from May to July. Cumulative dispersal rates were minimal (< 1%) prior to the planned start of the aerial survey (23 June) and increased to 11.2% by the planned survey completion date (10 July). The increased cumulative dispersal rate during the remainder of the observation period (end of July) suggests potential bias in surveys that occur beyond 10 July, however surveys past this date are rare (< 10% between 1973 and 2016). As a result, movements of mother-pup pairs during the pupping season are not likely to influence aerial survey estimates.

## Introduction

Precise measures of population size are necessary for demographic studies and for developing successful management and conservation strategies. For pinnipeds (seals, sea lions, and walrus), which have an aquatic lifestyle, direct counts of the entire population are often unfeasible because some proportion of the population may be at sea during a census (e.g. [1, 2]). To overcome these challenges, a subset of the population, such as newborn pups, can be counted and used as an index of population size or to track changes over time [3, 4].

For Steller sea lions (*Eumetopias jubatus*), which are widely distributed throughout the Pacific Rim from Japan to California, USA [4–6], there is substantial variation in population trends across the species’ range [4, 7]. The western distinct population segment, which includes breeding areas west of 144° W, is listed as “endangered” under the U.S. Endangered Species Act [8], whereas the eastern distinct population segment was recently removed from the list of threatened and endangered species [4, 9]. To monitor Steller sea lion population trends in Alaska, prior to 2005, counts of newborn pups were obtained by carefully walking through the rookery, which created considerable disturbance [10, 11]. Since 2005, pup counts have been conducted during annual aerial surveys, with some supplemental land- or boat-based counts [4, 10, 11]. The aerial pup count surveys occur between late June and mid-July when pups are approximately 1 month old [4, 12, 13].

The dates of aerial surveys are constrained by a number of factors, including the timing of pupping, weather, flight logistics, and the travel distance to cover the sea lions’ range. Sea lion behavior is also considered because surveys must be conducted after the majority of pups are born, yet before pups begin to spend significant time in the water [12–15]. Mean pupping date for Steller sea lions in Alaska generally occurs between 8 and 12 June, with the first births occurring in mid- to late May [12, 13]. Steller sea lion pups begin swimming at 2 to 4 weeks of age and can leave the rookeries with their mothers when 2 to 3 months of age [15–18]. Raum-Suryan et al. [18] reported sea lions as young as 2.5 months old traveled with their mother more than 120 km from the natal rookery.

After giving birth, female sea lions alternate short foraging trips to sea (~ 10 to 24 hr) with periods on land for nursing their young pup (~ 18 to 24+ hr) [19–21]. As a result, dispersal status cannot be determined based simply on sea lions leaving the breeding beaches. If mother-pup movements occur during the aerial survey period, pups may be missed or potentially counted more than once. Therefore, it is necessary to estimate Steller sea lion dispersal, defined here as permanent movements away from the pupping sites, during the reproductive period (May–July). To deal with the uncertainty of knowing true dispersal status and unknown detection probabilities, we used a multistate mark-recapture model with state uncertainty to estimate dispersal rates [22, 23]. Using data from a long-term study site that monitors Steller sea lion behavior and demographics, the multi-state mark-recapture framework allowed us to estimate the amount and timing of dispersal during the reproductive season and to quantify dispersal that may occur during the annual population census.

## Material and methods

### Data collection

**Fig 1:**
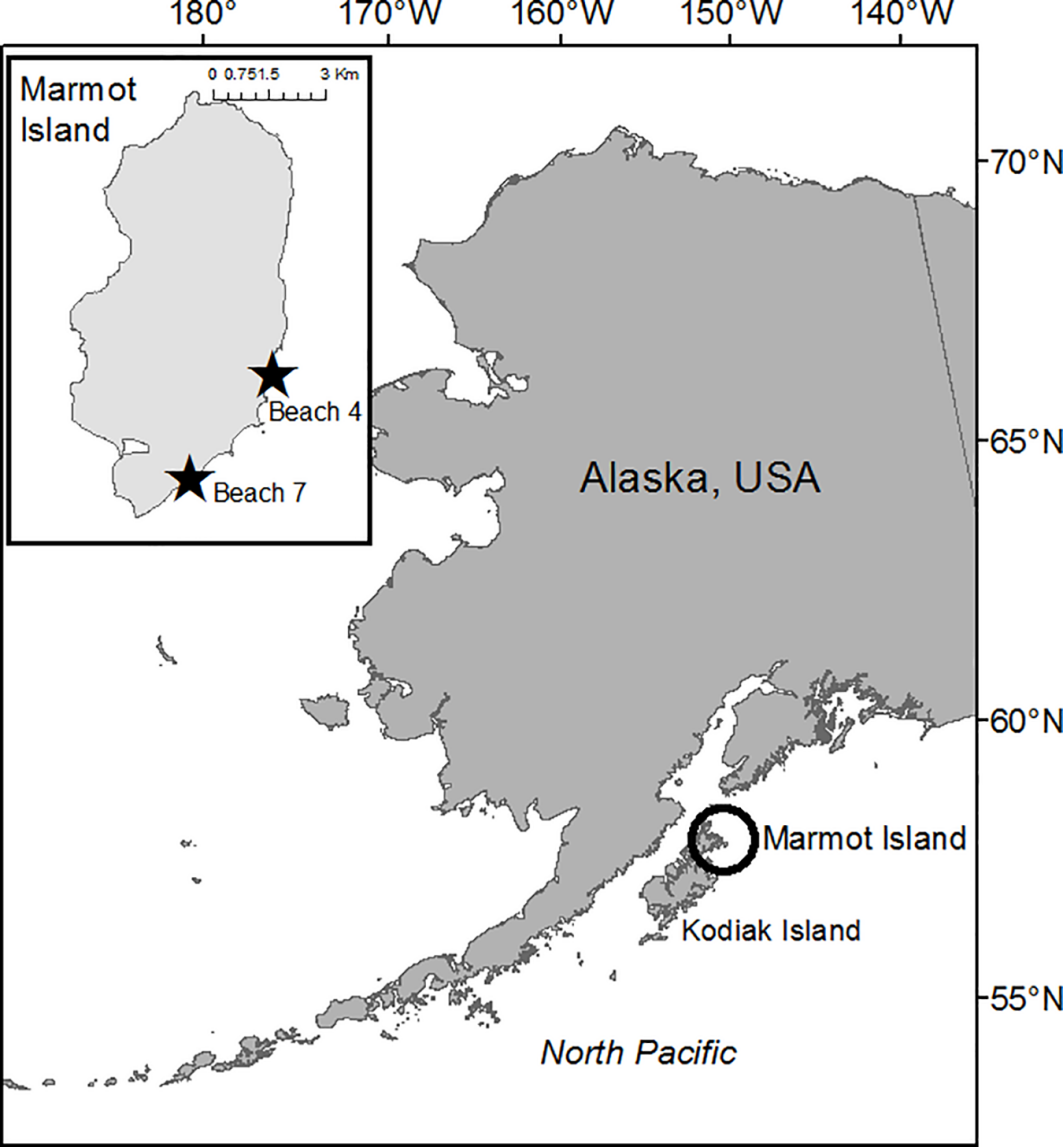
Location of Steller sea lion research sites on Marmot Island (Alaska, USA). Marmot Island (circle) is located 45 km northeast of Kodiak Island in the Gulf of Alaska. Inset: Visual observations of permanently marked Steller sea lions were recorded from cliff top overlooks at two rookeries, beach 4 and beach 7 (denoted by black stars), between 2000 and 2013.

Between 2000 and 2013, data were collected from land-based observation sites on Marmot Island, Alaska (USA, 58.22°N, 151.84°W), which is part of the Kodiak archipelago (Fig 1). Sea lions breed at two rookery beaches along the southeast side of the island, beaches 4 and 7 (Fig 1, inset). At beach 4, observations of the rookery were made daily, weather permitting, during June and July from 2000 to 2005 and between May and July from 2007 to 2013. At beach 7, prior to 2005, field teams were only able to visit one day a week (minimum); however, daily observations began when a permanent field camp was installed in 2005. During 2006 a research injunction resulted in a shortened field season (July only) and in 2003 only a single marked sea lion was observed. As a result these years (2003 and 2006) were not included in the dispersal analysis.

**Fig 2:**
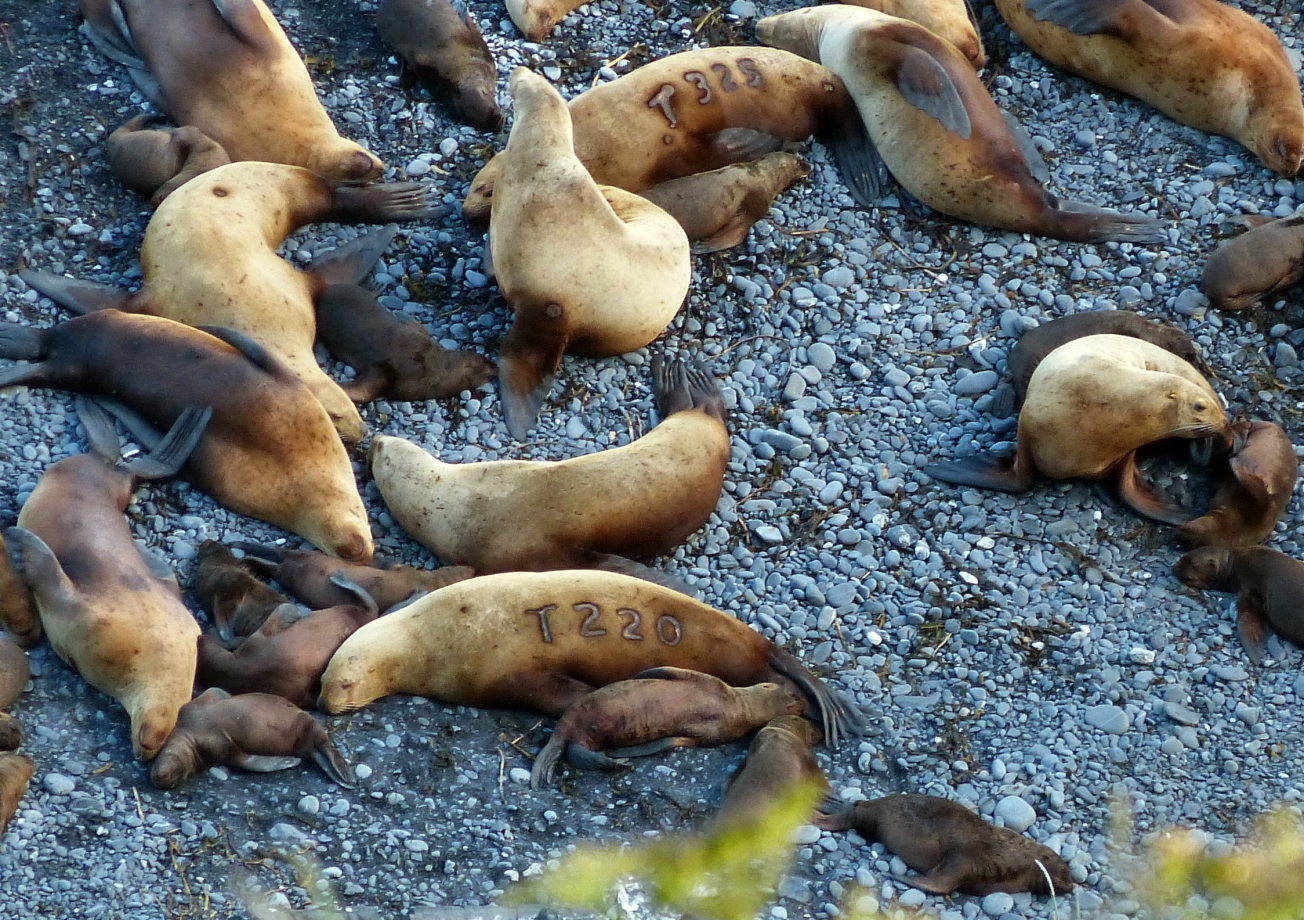
A group of Steller sea lion mothers and pups viewed from a resight location on Marmot Island. Two permanently marked adult females (T220 and T325) are visible with pups laying at their side.

From cliff edges approximately 50 to 300 m above the rookeries, observations of the rookery were conducted using binoculars or spotting scopes between 10:00 and 18:00 h (local time) [24]. Data collected included counts of sea lions by sex and age classes, recordings of sea lion behavior, and resighting of permanently-marked individuals. As part of an ongoing research program examining Steller sea lion survival and dispersal, permanent marks (hot-brands) are applied to Steller sea lions in Alaska by the National Marine Fisheries Service and Alaska Department of Fish and Game [24–28]. Steller sea lion pups were individually marked (hot-branded) at 2–5 weeks of age in late June or early July. Pups were marked by hot-branding the letter ‘T’ corresponding to the natal Marmot Island rookery followed by a unique 1- to 3-digit number starting on the left shoulder and extending down the left side (Fig 2) using the methods of [29]. All pups were weighed, measured (length), and immobilized using gas (isoflurane) anesthesia prior to branding. A minimum pup weight of 20 kg was established to avoid branding a very young animal shortly after birth. The total duration that an individual pup was handled (measuring and branding) was approximately 5 minutes. During the study period, approximately 100 pups/year were branded on Marmot Island in 2000, 2002, 2004, 2008 and 2010 [25, 30]. A total of 751 pups were also branded on Marmot Island from 1987 to 1988, some of which were observed during the study as 13+ yr old adult females with pups.

For each sighting of a marked animal, observers recorded the brand with a level of confidence, the sea lions’ age and sex, and the behavior of the individual. A detailed description of the data collection procedures can be found in [12, 24, 30]. For this analysis, only marked females associated with a pup (e.g. resting together touching, interacting, or nursing) and marked pups associated with a female (touching, interacting, or suckling) at least once during the field season were included in the analyses.

### Dispersal model

To estimate dispersal rate, we used a multi-state capture-recapture approach including state uncertainty (R package “marked,” v. 1.1.13, [31]) through a Hidden Markov Model (HMM) formulation [22, 23]. The capture-recapture model is composed of three submodels, 1) the state transition model, 2) the detection model, and 3) the observation model. The state model describes how the animal transitions from one state to another between observations. For this analysis, the two states for sea lions were “home” and “away” (i.e., permanent dispersal occurred). The state model describes the probabilities of a sea lion being “home” or “away” on an observation occasion given that it was “home” on the last occasion. The “away” state was defined as permanent movement to another location for the duration of the monitoring period, which could include either movement to the alternative non-home beach on Marmot Island, or movement away from the island altogether. Because we are defining the “away” state as a permanent transition, the probability of returning to the “home” state is zero. The detection portion of the model describes the probability that an animal is seen on each occasion given that it is either “home” or “away”. Finally, the observation model describes the probability of accurately classifying the state given the animal was observed. For example, if the sea lion is detected on the home rookery, then the “home” state is assigned with certainty (by definition), however, if the animal is observed away from the home rookery, the observer does not know (at that time) if the animal has permanently moved, i.e., transitioned to the “away” state, so it is recorded as “unknown”. The probability of observing an “unknown” state while the animal is still in the “home” state is the observation model parameter. The probability of observing “unknown” if the animal is in the “away” state is 1 by definition (it will always be observed away from the home rookery after dispersal).

Adult female sea lions were assigned a “home” beach based on the location they were first observed with a pup. The home beach for marked pups was assigned as the branding location (i.e., beach 4) for all years. Each sea lion was assigned a daily observation: home (“h”), not observed (“0”), and unknown (“u”). As an example, consider the following hypothetical daily resight histories:

#1: h u h u h u h u h
#2: h h h h u 0 u u u
#3: h h h h 0 0 0 0 0

Sea lion #1 alternated between the home beach and an alternate beach on successive days. For even days the model categorized Sea lion #1 as being in an unknown state because it was observed away from the rookery, but it was unknown if the move was permanent. Ultimately, the model assigned Sea lion #1 to a “home” state throughout the observation history because it was observed at home on the last day. Sea lion #2 was seen at home on the first four days and at another beach on four of the last five observation days. The model determines there is a high probability that Sea lion #2 moved to the “away” state because it was not observed at home after day 4. For Sea lion #3, the model would also determine there is a high probability that the animal moved to the “away” state because it was not observed at home after day 4.

When multiple sightings occurred within a day, a sea lion was considered “home” if at any point during the day it was observed at the home beach. All analysis was conducted within a single year (season). Therefore, branded individuals observed in multiple years were considered independent observations (range: 2–9 resight years per adult sea lion).

To account for Steller sea lion behavior, the detection probabilities varied between states and age classes, based on physical and behavioral differences, which include brand size/readability, activity level while on land, and time in the water. For the “home” state, we assumed a quadratic change in detection over the season with a fixed slope among years. The detection change over the pupping season took into account the decline in visit duration for nursing adult females [21, 32] and also the increase in water activity by pups [15, 18, 33]. The varying intercept of the detection model allowed for annual variation in observation quality, resulting from weather or observer biases. Because less data were available for the “away” state, we simplified the detection model by removing the time component and letting only the intercept vary among years. Finally, on days where no resight effort occurred detection was fixed at zero. After giving birth, females can spend up to 10 days of the post-partum period onshore [20, 32], therefore, dispersal was closed during the first 10 days of the season (May 24 to June 2). This only affected the later study years (2008–2013) when the field season started in May.

The transition probability from “home” to “away” was modeled with a linear daily trend on the logit scale. Survival was set to one for adults over the course of the breeding season; however, pup survival was allowed to be less than one during the breeding season. During the observation period, pups are dependent upon their mother for survival and therefore any dispersal should be equal for the two age classes. As a result, we chose to combine the two groups for the dispersal analysis. To examine if there were annual differences in dispersal rates, we tested the model with years separately and pooled and selected the model with the lowest Akaike information criterion (AIC). Confidence intervals (95%) for daily dispersal rates and cumulative dispersal rates were computed using a parametric bootstrap by assuming parameters were approximately normally distributed on the logit scale. Means are presented ± SE.

All work was conducted in accordance with and under the authority of the U.S. Marine Mammal Protection Act (National Marine Fisheries Service, NMFS Permits #782–1532, 782–1768, 782–1889, and 14326). The Marine Mammal Protection Act was established in 1972 requiring all research conducted on marine mammals in the United States be done under the authority of federal permits issued by either NMFS or U.S. Fish and Wildlife Service (USFWS). All applications for a permit to conduct research on marine mammals have gone through a four-stage review process that includes: 1) agency review (either NMFS or USFWS); 2) a public notice and review period; 3) review and recommendation from the Scientific Advisers to the U.S. Marine Mammal Commission; and 4) a final action by the reviewing agency. All research activities described in this manuscript have gone through and been approved by this process. At the time when the research was conducted by NMFS there was no additional requirement for review of these procedures by an institutional review board or ethics committee. A NMFS Institutional Animal Care and Use Committee was established by the Alaska Fisheries Science Center in 2010 and protocols described here were reviewed and approved by this committee (IACUC # A/NW 2010–4).

## Results

### Data collection

Field seasons averaged 53.4 ± 0.9 d (range: 28–65 d) and sighting effort was 44 ± 1.0 d per year (range: 21–59 d, Table 1). The earliest start date for the resight effort was 24 May in 2013 and the latest end date was 31 July in 2007. Over all study years, 586 brand resights were included in the model (Table 1). Between 86% and 100% of branded pups were resighted while associating with a female in each branding year (brands applied: 2000 = 107, 2002 = 89, 2004 = 75, 2008 = 85, and 2010 = 78, Table 1). The number of branded adult female sea lions that were associated with a pup ranged from one in 2000 to 38 females in 2013 (Table 1).

**Table 1:** Summary of brand resight effort and results from Marmot Island, AK, between 2000 and 2013. Start date is the first day of observations in each year and end date is the last. Field season is the number of days between the start and end date. Resight days are the number of days in each year that observers were at the rookery. Only permanently marked pups observed associated with a female (Pups, see methods) and permanently marked adult females (Adult females) observed with pups were included in the analysis.

Year	Start date	End date	Field season (d)	Resight days	Pups	Adult females
2000	June 25	July 23	28	21	92	1
2002	June 12	July 27	45	30	78	2
2004	June 1	July 28	57	42	70	3
2005	June 11	July 22	41	38	0	7
2007	May 27	July 31	65	53	0	18
2008	May 29	July 27	59	42	83	15
2009	May 28	July 26	59	53	0	26
2010	May 28	July 28	61	59	78	24
2011	May 27	July 25	59	54	0	24
2012	May 27	July 25	59	53	0	27
2013	May 24	July 24	61	54	0	38

### Dispersal model results

**Fig 3:**
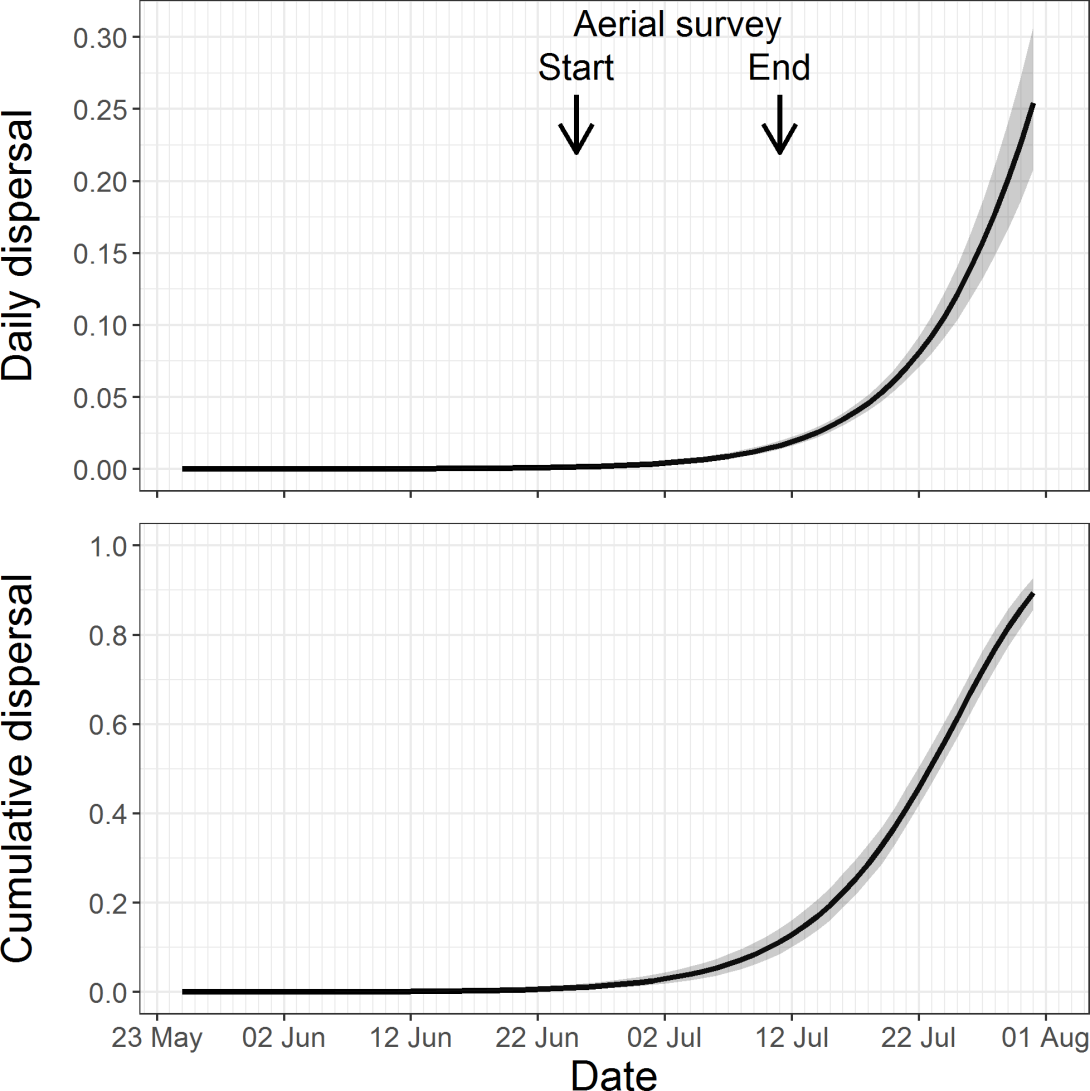
Estimated daily (top) and cumulative (bottom) dispersal for marked Steller sea lions on Marmot Island, AK, between 2000 and 2013 (Table 1). Black lines denote mean dispersal for all study years and shaded areas encompass the 95% confidence intervals. The planned start of the annual aerial survey to measure pup production is 23 June and the end is 10 July, denoted by black arrows.

Interannual differences in dispersal rate were not supported (AIC: 22758.94 vs. 20628.77); therefore, year effects were removed from the model. Daily dispersal rates increased as the pupping season progressed, peaking at 25.4% ± 2.5% (95% CI: 20.8–30.7%, Fig 3 top) on 31 July, the end of the analysis period. As a result, cumulative dispersal through 31 July reached 89.4% (95% CI: 85.7–92.8%, Fig 3 bottom). Prior to the planned start of aerial surveys (23 June), the cumulative dispersal rate was low (0.9%; 95% CI: 0.5–1.4%). Within the planned aerial survey period (between 23 June and 10 July), cumulative dispersal increased to 11.2% (95% CI: 8.5–14.2%). In some years, aerial surveys continued until 16 July [34], and in those extra survey days cumulative dispersal increased to 25.4% (95% CI: 21.8–29.6%). Based on the modeled cumulative dispersal rate, using 2013 pup counts for Marmot Island beach 4 (291 pups, [12]), we would expect that prior to the aerial survey period only 1 to 4 mother-pup pairs would have moved away from the home rookery. By the end of the aerial survey period this number increased to 24–41 mother-pup pairs.

## Discussion

Movements of Steller sea lion mother-pup pairs have been previously reported during the pupping season [24, 35]; however, the amount of dispersal has not been quantified. By using observations of marked sea lions collected from a long-term study site, we were able to estimate the extent of these movements. This quantification is necessary to understand how or if dispersal during the pupping season impacts counts of Steller sea lions that are used for annual population estimates [4, 10, 11].

Steller sea lion females must feed during the nursing period, and as a result, dispersal cannot be estimated simply by counting animals leaving the rookery [19–21]. Therefore, it was necessary to use the multi-state capture-recapture approach including state uncertainty to estimate dispersal rates [22, 23]. When possible model parameterization was selected based on available biological data. For example, Steller sea lion pups are thought to be dependent on their mothers for approximately a year or longer [16, 18, 36]; therefore, we expected dispersal between age classes would be identical as mother-pup pairs are required to stay together for pup survival. For other parameters, such as detection probabilities, the detection curve over time for the home state was parameterized based on known seasonal changes in behavior for both adult females and pups [15, 20, 37]. For example, adult female foraging trip lengths increase and for pups, time spent in the water increases over the pupping season, which both reduce detection. Kaplan et al. [38] reported daily resight probabilities for Steller sea lion pups up to 3 weeks of age declined over time based on a quadratic relationship and there was variation between years. Adjusting of model parameters, such as through the addition of more information about female attendance patterns or increased data about the “away” state would be valuable to fine-tune the dispersal estimates.

On Marmot Island, mother-pup dispersal patterns were found to be consistent among years, and cumulative dispersal rates were minimal (< 5%) for the month of June (Fig 3). Prior to the planned start of the aerial survey (23 June), cumulative dispersal was less than 1%. However, dispersal began to increase during the preferred survey period (23 June to 10 July), exceeding 10% by the end of the survey period. During some years, the aerial surveys could not be completed by 10 July and extended as late as 16 July [34]. In these cases the estimated cumulative dispersal would have been 25% for rookeries surveyed on 16 July. Our model results for pupping season dispersal are similar to reports of Steller sea lion movements at the Forrester Island Complex, AK, and earlier studies at Marmot Island [24, 27, 35]. Hastings et al. [35] reported a small percentage of relocations of marked pups and marked adult females occurred between 5 July and 20 July within the island complex, whereas Chumbley et al. [24] reported a rapid decline in counts of pups at two study beaches around 10 July, although there was variability among years [24].

The estimated dispersal rates for mid-late July suggest aerial survey counts obtained outside the preferred survey window could be negatively biased. However, the mid-July movements are likely localized due to the limited swimming distances of pups and may entail mother-pup pairs moving to other nearby areas [15, 35], such as moving between beaches on an island or within islands of a larger breeding complex. For example, Hastings et al. [35] reported movements between islands within the Forrester Island Complex with mother-pup pairs traveling less than 5 km between 5 July and 20 July. If localized movements are driving dispersal prior to 10 July, then there would be a negligible effect on pup counts obtained during aerial surveys because Marmot Island is treated as a single rookery for the purposes of reporting pup counts. Further, for Steller sea lion population trends, analyses are conducted on larger scales such as rookery clusters areas or regions (e.g., eastern Gulf of Alaska vs. central Gulf of Alaska) [4, 39]. Finally, surveys after 10 July are not common. Between 1973 and 2016, 91% of all pup counts used in population trend analyses occurred between 23 June and 10 July (none prior), with a median survey date of 30 June, when cumulative dispersal was less than 5% [40].

The cause for dispersal of mother-pup pairs during the pupping season is unknown, but could result from a number of factors including changing beach conditions (e.g., crowding or increased storm/wave surges), individual “preference”, or the need to move to areas with greater prey availability. As Steller sea lion pups grow, the rate of milk intake increases exponentially [41]. This may lead females to move to areas of increased prey resources or decrease competition from other nursing females to sustain increasing milk production [26]. In addition, these movements may also provide pups better access to resources as they begin to supplement their suckling with some prey capture [18, 36, 42]. Regardless of the reason for these movements, data from Marmot Island indicated movements of Steller sea lion mother-pup pairs either occurred outside the preferred aerial survey windows used for population assessments or had minimal effect on aerial survey results.

This study demonstrated the use of a multi-state mark-recapture framework to estimate the amount and timing of dispersal during the pupping season for Steller sea lions, but this framework could be applied to other species or systems. For example, movements of mother-pup pairs during the early dependency period have also been reported for California (*Zalohpus californianus*), Australian (*Neophoca cinerea*), and New Zealand sea lions (*Phocarctos hookeri*) [43–45]. In each of these species, the extent of these movements and potential impacts on population estimates are considered minimal, but have not been fully quantified [43–45]. For California sea lions, aerial surveys are used to estimate pup production on the West Coast of the United States, with surveys occurring in July [46]. The report of mother-pup dispersal in this species was from a neonate (< 4 weeks old) which occurred prior to the first week of July from a breeding colony in Mexico [45]. Although this report was for a single mother-pup pair, it shows that dispersal of mother-pup pairs is possible prior to population surveys. For Australian and New Zealand sea lions aerial surveys are not used, but movements of mother-pup pairs prior to the pup production census could result in missed pups and a biased population estimate. Finally, this framework is not limited to dispersal within a reproductive period but could also be applied to more long-term movements of individuals. For example, although many pinnipeds display strong breeding site fidelity [47], multi-year analyses of resight data could be used to quantify permanent movement of animals to new breeding locations (e.g. [48]) or different populations segments (e.g. [49, 50]).

## Supporting information

S1 Table Steller sea lion brand resight dataset used for dispersal analysis. Brand is the permanent marking on each sea lion, Home_beach is the location where pups were branded or adult females were first observed with a pup (see Fig 1), Age is the age of the marked individual (P = pup, A = adult female), Start_date is the model start for each year (24 May), and Resight_history is a character string of daily resight categories (0 = not resighted, H = resighted at home beach, U = resighted at beach other than home beach).
(CSV)
